# The PD-1/PD-L1 Axis in the Biology of MASLD

**DOI:** 10.3390/ijms25073671

**Published:** 2024-03-25

**Authors:** Rosaria Maria Pipitone, Giulia Lupo, Rossella Zito, Ayesha Javed, Salvatore Petta, Grazia Pennisi, Stefania Grimaudo

**Affiliations:** Department of Health Promotion, Mother and Child Care, Internal Medicine and Medical Specialties, University of Palermo, Piazza delle Cliniche, 90127 Palermo, Italy; giulia.lupo03@unipa.it (G.L.); rossella.zito@unipa.it (R.Z.); ayesha.javed@unipa.it (A.J.); salvatore.petta@unipa.it (S.P.); grazia.pennisi@unipa.it (G.P.)

**Keywords:** MASLD, MASH, PD-1, PD-L1, HCC, CD8^+^ T cells exhausted, T Reg cells, cytotoxic lymphocytes

## Abstract

Metabolic Dysfunction-Associated Steatotic Liver (MASL), previously named nonalcoholic fatty liver (NAFL), is a multifactorial disease in which metabolic, genetic, and environmental risk factors play a predominant role. Obesity and type 2 diabetes act as triggers of the inflammatory response, which contributes to the progression of MASL to Metabolic Dysfunction-Associated Steatohepatitis and the development of hepatocellular carcinoma. In the liver, several parenchymal, nonparenchymal, and immune cells maintain immunological homeostasis, and different regulatory pathways balance the activation of the innate and adaptative immune system. PD-1/PD-L1 signaling acts, in the maintenance of the balance between the immune responses and the tissue immune homeostasis, promoting self-tolerance through the modulation of activated T cells. Recently, PD-1 has received much attention for its roles in inducing an exhausted T cells phenotype, promoting the tumor escape from immune responses. Indeed, in MASLD, the excessive fat accumulation dysregulates the immune system, increasing cytotoxic lymphocytes and decreasing their cytolytic activity. In this context, T cells exacerbate liver damage and promote tumor progression. The aim of this review is to illustrate the main pathogenetic mechanisms by which the immune system promotes the progression of MASLD and the transition to HCC, as well as to discuss the possible therapeutic applications of PD-1/PD-L1 target therapy to activate T cells and reinvigorate immune surveillance against cancer.

## 1. Introduction

Metabolic Dysfunction-Associated Steatotic Liver Disease (MASLD), previously named Nonalcoholic Fatty Liver Disease (NAFLD), is the most chronic liver disease due to the associated complex metabolic disorders and the high risk of complications, such as liver decompensation, hepatocellular carcinoma (HCC), and cardiovascular diseases [1,2,3]. Indeed, until a few years ago, NAFLD was considered the hepatic manifestation of Metabolic Syndrome (MetS) in terms of insulin resistance (IR), atherogenic dyslipidemia, abdominal obesity, and hypertension [4,5]. Recently, its terminology has been revised and updated, considering NAFLD a key factor in MeS [6], associated with metabolic disorders, such as obesity and type 2 diabetes (T2D) [5,7]. All these aspects led to revising the definition of NAFLD and considering it a metabolic disease. For this reason, it has recently been renamed Metabolic Dysfunction-Associated Steatotic Liver Disease (MASLD) and Metabolic Dysfunction-Associated Steatohepatitis (MASH). In addition, the coexistence of metabolic and alcoholic liver disease has been identified, and the requirement of the absence of excessive alcohol consumption has been eliminated from its definition [4]. This point it is very important because the alcoholic parameter is measured through questionnaires and, often, is reported by individual with MASLD in a highly variable manner, from zero or near-zero to sporadic consumption during “social drinking” [4].

Worldwide, MASLD has affected 30% of the population [7]. In the next few years, its prevalence and the associated healthcare costs are expected to increase due to the spread of obesity and diabetes [3].

The multifactorial pathogenesis in which metabolic, genetic, and environmental risk factors exist has been widely explored, especially on the steatogenic and fibrogenic sides. Liver inflammation involved in the onset and progression of MASLD has aroused great interest. Evidence supports the key role of the immune response in leading to fibrogenesis promoting MASH and HCC development [8,9,10].

In MASLD, obesity and diabetes act as triggers of the inflammatory response: IR and lipotoxicity activate the hepatic immune system, and the persistent pro-inflammatory state contributes to fibrogenesis and carcinogenesis [11].

The liver hosts several resident immune system cells, such as Kupffer cells (KCs), natural killer cells (NK cells), natural killer T cells (NKT cells), mucosal-associated invariant T cells, γδ T cells, conventional CD8^+^ T and CD4^+^ T cell subsets (Th1, Th2, Th17, and T Reg cells) [12].

Different regulatory pathways counterbalance the activation of the immune system. Between them, the programmed cell death protein 1 (PD-1) represents an immune checkpoint surface receptor, acting as a key regulator of T cells and macrophage responses during chronic inflammation and cancer [13].

PD-1 activation keeps the peripheral immune tolerant and promotes the immune escape from cancer. The aim of this review is to illustrate the main pathogenetic mechanisms by which the immune system promotes the progression of MASLD and the transition to HCC and to discuss the possible therapeutic applications of PD-1/PD-L1 target therapy.

## 2. Innate and Adaptative Immunity in the MASLD

The immune system is essential in maintaining liver homeostasis and responding to inflammatory stimuli. The hepatic immunological environment involves a complex multistage network and interactions established between liver-resident cells and peripheral leukocytes. In this context, innate and adaptative immune mechanisms support hepatic inflammation in MASLD development and progression. However, it is largely unexplored how immune responses link with MASLD pathogenesis, but, to date, lobular inflammation is considered the driving force for MASH, fibrosis, cirrhosis, and HCC development.

### 2.1. The Major Immune Cell Types in the Phisiological Status and in the Early Stage of MASLD

Hepatic innate immune cells consist of a large population of macrophages, such as resident KC and lymphocytes such as NK and NKT cells, while adaptive immunity includes humoral immunity mediated by B cells and cellular immunity mediated by T cells [11,14].

**Figure 1:**
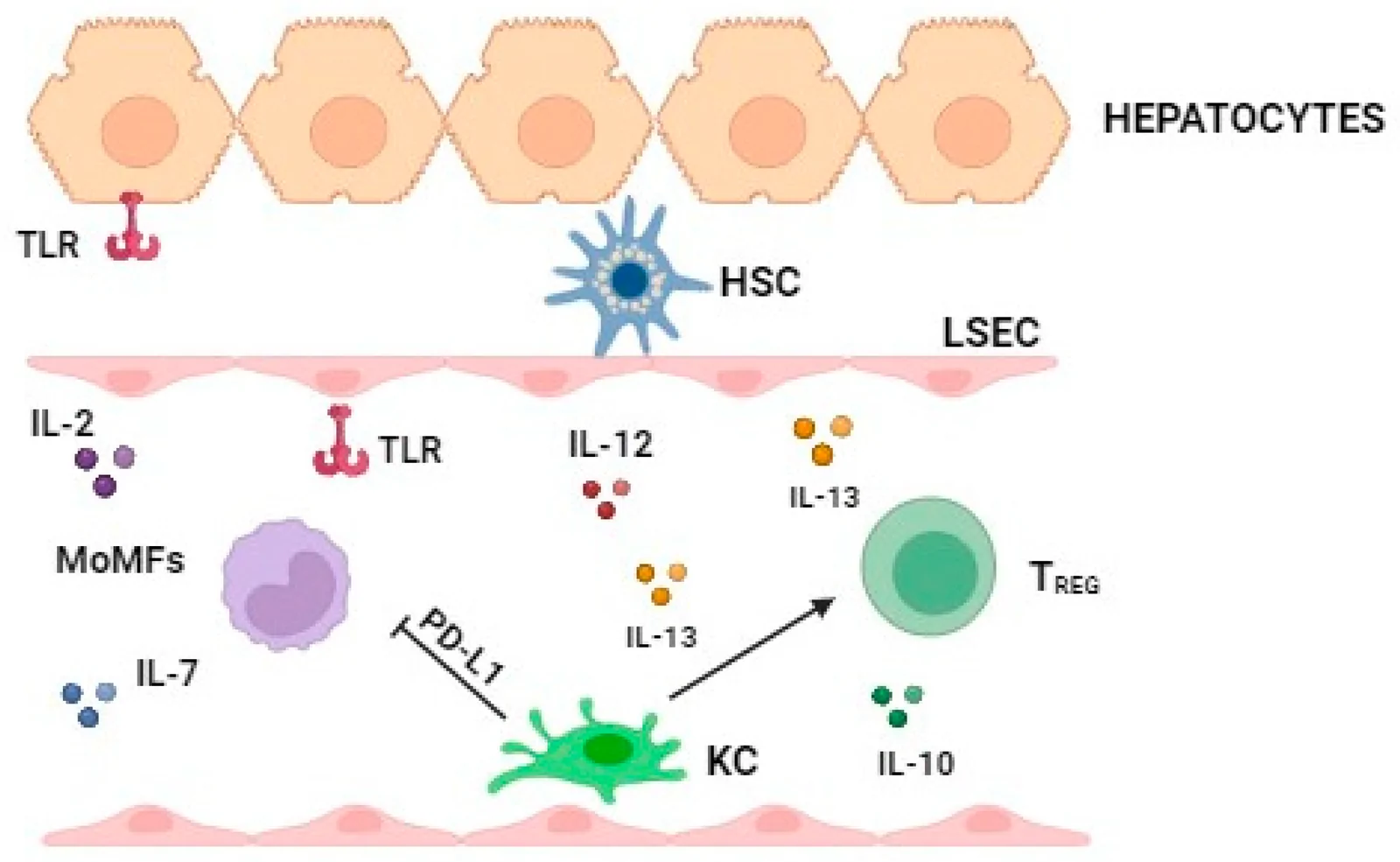
Schematic representation of physiological liver immunity status. In the healthy liver, the immunological homeostasis is maintained through the balance between basal pro-inflammatory cytokines, such as IL-2, IL-7, IL-12, and anti-inflammatory cytokines, like IL-10 and IL-13, and by the expression on PD-L1.Parenchymal and nonparenchymal cells, such as hepatocytes, HSCs, monocytes, KCs, and LSECs, contribute to maintaining liver immunity homeostasis cross-talk with T Reg cells.

Both B and T cells maximize immune surveillance, participating in maintaining tissue homeostasis and inducing systemic tolerance. The hepatic immunological tolerance involves complex and multistage interactions between liver-resident cells and peripheral leukocytes (Figure 1).

In a healthy liver, in response to acute tissue injury, the initial phase of hyper-inflammation is followed by its resolution and by anti-inflammatory phases.

Under physiological conditions, immunological homeostasis is maintained by the hepatic cytokine milieu, the balance between basal pro-inflammatory cytokines and anti-inflammatory cytokines [15], and parenchymal and nonparenchymal cells, like hepatocytes, hepatic stellate cells (HSCs), and liver sinusoidal endothelial cells (LSECs) (Figure 1) [16]. In this context, monocyte-derived cells can differentiate into liver DCs or monocyte-derived macrophages (MoMFs). The latter do not contribute to the pool of local resident macrophages but, together with KCs, which represent the “self-renewing pool”, play a role in MASLD progression, modulating hepatocyte fate (Figure 1) [17].

In the early stage of MASLD, the excessive hepatic accumulation of lipid, triggers the release of Damage-Associated Molecular Patterns (DAMPs), which are recognized by Toll-Like Receptors (TLRs), mainly TLR 1–5, expressed on the hepatic cells. It has been reported that, in MASLD patients, the expression of these TLRs is significantly upregulated [18]. These receptors are the primary sensors detecting DAMPs and triggering the activation of pro-inflammatory pathways, such as NF-κB and mitogen-activated protein kinase (MAPK) [19].

However, the concomitant state of lipid overload into hepatocytes, IR, and lipotoxicity establishes chronic inflammation, characterized by constant activation of immune responses, without adequate resolution of inflammation [20].

KCs, the hepatic resident macrophages, represent the first responders to the inflammatory stimuli in MASLD [21]. Specifically, the expression of TLR4 on KCs has been shown to be increased in MASLD patients with a diagnosis of fibrosis [22].

KCs are also involved in MASLD progression, through the release of pro-inflammatory cytokines, triggering a crosstalk with hepatic sinusoid endothelial cells, HSCs, neutrophils, monocytes, T cells, and dendritic cells (DCs) [23]. It has been reported that KCs upregulate ICAM-1 to attract more neutrophils into the liver; indeed, the serum ICAM-1 level was significantly higher in the patients with MASH than in the patients with simple steatosis and in the normal subjects [24]. In addition, KCs acting as an APC, play a tolerogenic role by expressing Programmed cell Death 1-Ligand 1 (PD-L1), an inhibitory molecule that engage PD-1, inhibits T cell-mediated immunity, and induces the activity of T Reg cells; the last cells protect against deregulated immunity responses [25]. On the other hand, KCs, during extensive injuries, increase the MoMFs influx, which replaces the native macrophages and activates naïve CD4^+^ T cells [26].

NK cells, one of the major populations of hepatic lymphocytes, representing about 30–50% of them, play a crucial role in the development of hepatic inflammation, antiviral responses, and tumor surveillance, through their cytolytic activity and the release of immunomodulatory cytokines. Furthermore, NK cells can kill activated HSCs, in an NKG2D-retinoic acid-dependent manner, thereby improving liver fibrosis [27].

It has been reported that, in obese subjects, there is a significant increase in NK cells with an activated phenotype and impaired ability to release granules and cytokines, which may contribute to the liver fibrosis development in MASH patients [28].

Natural killer T cells (NKT), the innate-like lymphocytes, are characterized by the expression of both T and NK cell surface markers [29]. NKTs also have a role in hepatic lipid metabolism, activating, via the expression of CD1d, a lipid antigen receptor [30]. It has been observed that CD1d deficiency impairs metabolic parameters and induces hepatic steatosis [31]. Furthermore, during acute liver inflammation, activated by CD1d, NKTs play an immunoregulatory role through the secretion of IL-4, TNF-α, and INF-γ [32]. During the early stage of MASLD, it has been shown that the number of NKTs is reduced; however, in patients with moderate-to-severe steatosis, it an increase in NKTs has been shown, with a pro-inflammatory phenotype able to promote liver fibrosis via osteopontin production [33]. So the liver attempts to trigger inflammatory response and repair mechanisms, but at the same time, the inflammation drives disease progression [34,35].

### 2.2. Role of the Major Immune Cell Types in the Late Stage of MASLD

Growing evidence indicates that APCs, supported by neutrophils, are a critical component of innate and adaptative immune mechanisms, which are able to activate naïve T cells. This role is not only played by professional APCs, such as LSEC, KCs, and HSCs, but also by hepatocytes [36,37].

The neutrophils contribute to tissue damage, through the production of toxic molecules, such as proteases, ROS, cytokines, and Neutrophil Extracellular Traps (NETs) [38,39,40]. The last are active participants in the inflammatory environment responsible for MASLD progression to MASH; their generation is independent of neutrophil density but is correlated with their activated state [41].

Subsequently to neutrophils, macrophages and monocytes are rapidly recruited into the liver [42].

Initially, macrophages mediate NET efferocytosis, restoring tissue homeostasis after tissue injury; secondarily, they generate an inflammatory milieu that leads to excessive neutrophil activation and NETosis induction [43].

NETs are areas dense in IL-1β and IL-17A cytokines that contribute to inflammation and correlate with MASLD progression. In this context, IL-17A can activate HSC and promote fibrosis [44]. Furthermore, it has been reported that NETs are involved in MASH-associated HCC development through the generation of immunosuppressive microenvironments. It has been demonstrated that NETs promote T Reg differentiation via the metabolic reprogramming of naïve CD4^+^ T-cells, promoting tumor development by suppressing cancer immunosurveillance [45].

Clinical evidence has reported the effect of NETs on the progression of MASH to HCC. Specifically, elevated levels of NET markers, such as circulating Myeloperoxidase (MPO)-DNA complexes, have been found in the serum of patients with a histological diagnosis of MASH compared to patients with normal liver histology. 

**Figure 2:**
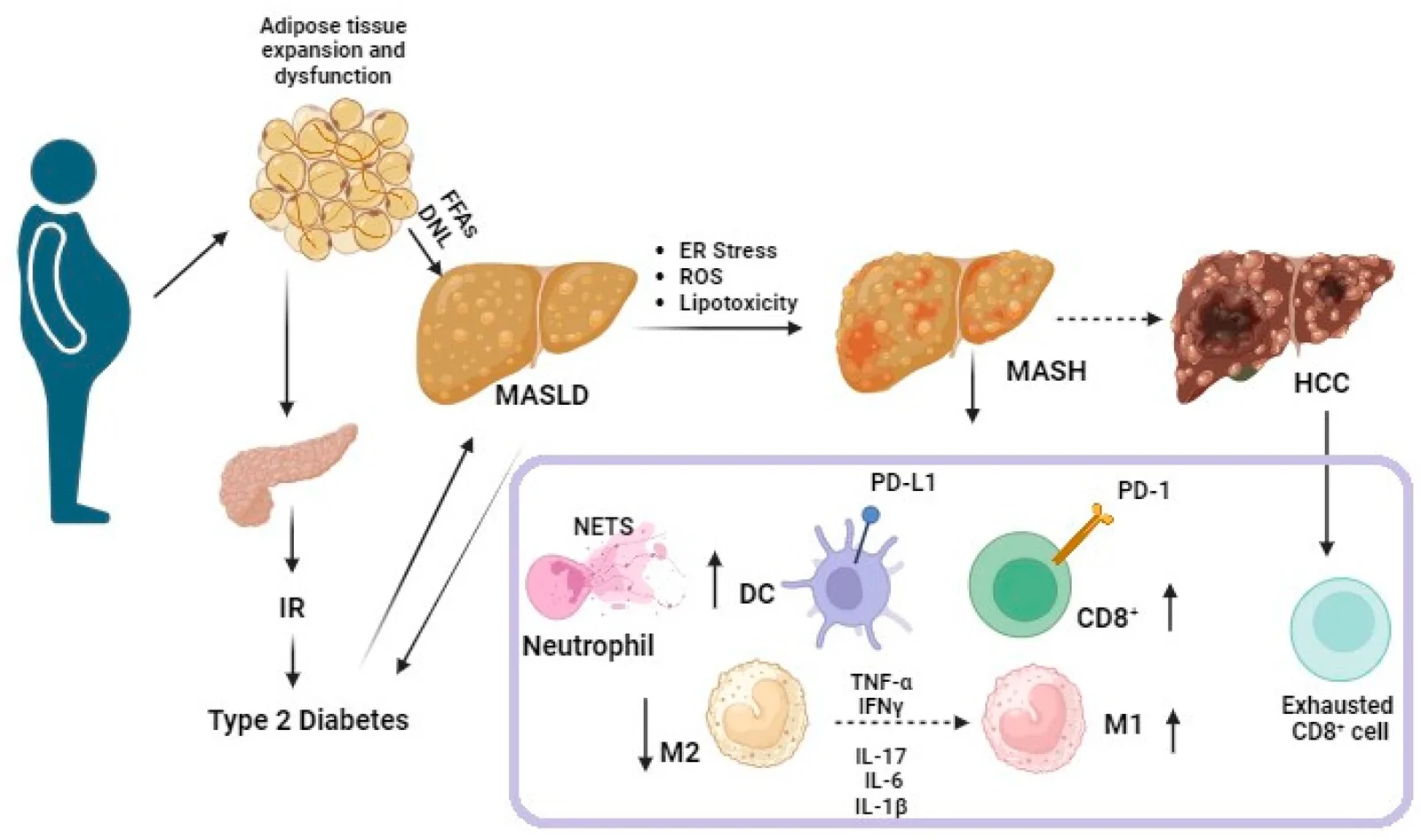
Schematic representation of MASLD pathogenesis. Type 2 diabetes and obesity in adipose tissue promote a low state of chronic inflammation and an excessive release of free fatty acids (FFAs). The excessive uptake from FFAs into the liver activates the de novo lipogenesis (DNL) to convert the excess of free fatty acids (FFAs) and glucose into triglycerides (TGs). The high lipid content accumulates into hepatocytes, resulting in innate immunity activation and toxic molecules production, such as ROS, cytokines, and Neutrophil Extracellular Traps (NETs). The continuous chronic inflammatory environment activates the adaptative immune response, inducing the increase in T Reg, T CD8^+^ cells, the polarization of macrophages into the M1 phenotype, and the production of inflammatory cytokines, such as IFN-γ, TNF-α, IL-1, IL-6, and IL-1β. This persistent chronic inflammation occurs in all stages of MASLD progression, mostly in NASH, and during the transition to HCC, this inflammatory chronic status is also responsible for T CD8^+^ cells differentiating into exhausted T lymphocytes.

During MASLD progression, in addition to neutrophils, the macrophagic population is fully activated, together with T Reg and cytotoxic CD8^+^ lymphocytes (Figure 2) [46]. It has been reported that the inhibition of hepatic macrophages can attenuate inflammation and the severity of liver damage, underling the crucial role of these cells in liver disease progression [47].

In the injured liver, it has been reported that monocytes can differentiate into inflammatory, angiogenic, and fibrogenic macrophages and can activate HSC or other precursor cells mediating the progression of MASH to fibrosis [48].

In this context, continuous regenerative response leads to the chronic activation of HSCs, collagen deposition, fibrosis, and subsequently cirrhosis with a switch from the fibrotic phenotype to the pro-carcinogenic one (Figure 2) [49].

### 2.3. Role of the Adaptative Immune Response in MASH Progression

Recent evidence suggests a main role of adaptive immunity in MASH progression since lymphocytic infiltration has been frequently observed in liver biopsies of MASH patients, often as focal aggregates of T cells, similar to ectopic lymphoid structures [9,50,51].

It has been reported that MASH is characterized by the hepatic lobular infiltrate cellular, represented mainly by of B cells, CD4^+^ cells, and CD8^+^ cells [9].

#### 2.3.1. The Humoral Immunity: B Lymphocytes

B cells comprise two cellular subpopulations: B1, which acts through the secretion of natural antibodies, and B2, which acts through the release of high-affinity antibodies [52].

The role of hepatic B cells is not completely understood, since they comprise only 5% of human intrahepatic lymphocytes in the healthy liver. Bruzzi S and colleagues have reported that B cells are detectable in a 63% liver biopsy from patients with MASH within inflammatory infiltrates, and it has been reported that B-cell depletion ameliorated the hepatic inflammatory response status [9,53,54].

After their stimulation, B2 cells are activated and express high levels of B Cell Activating Factor (BAFF or CD257); its receptor, BAFF-R, a tumor necrosis factor family member, is expressed on the surface of monocytes, macrophages, dendritic cells, neutrophils, and activated T cells; it is responsible for their activation [55]. In mouse models of MASLD, BAFF neutralization has been able to reduce steatohepatitis [53].

It has been reported that MASH patients have higher circulating levels of BAFF compared to those with simple steatosis. It has been suggested that serum BAFF levels could be useful for distinguishing MASH from simple steatosis. Indeed, it has been reported that, in MASH patients, an increase of 1.5 times the BAFF levels is associated with the presence of hepatocyte ballooning and advanced fibrosis [56].

#### 2.3.2. The Cellular Immunity: T Lymphocytes

**Figure 3:**
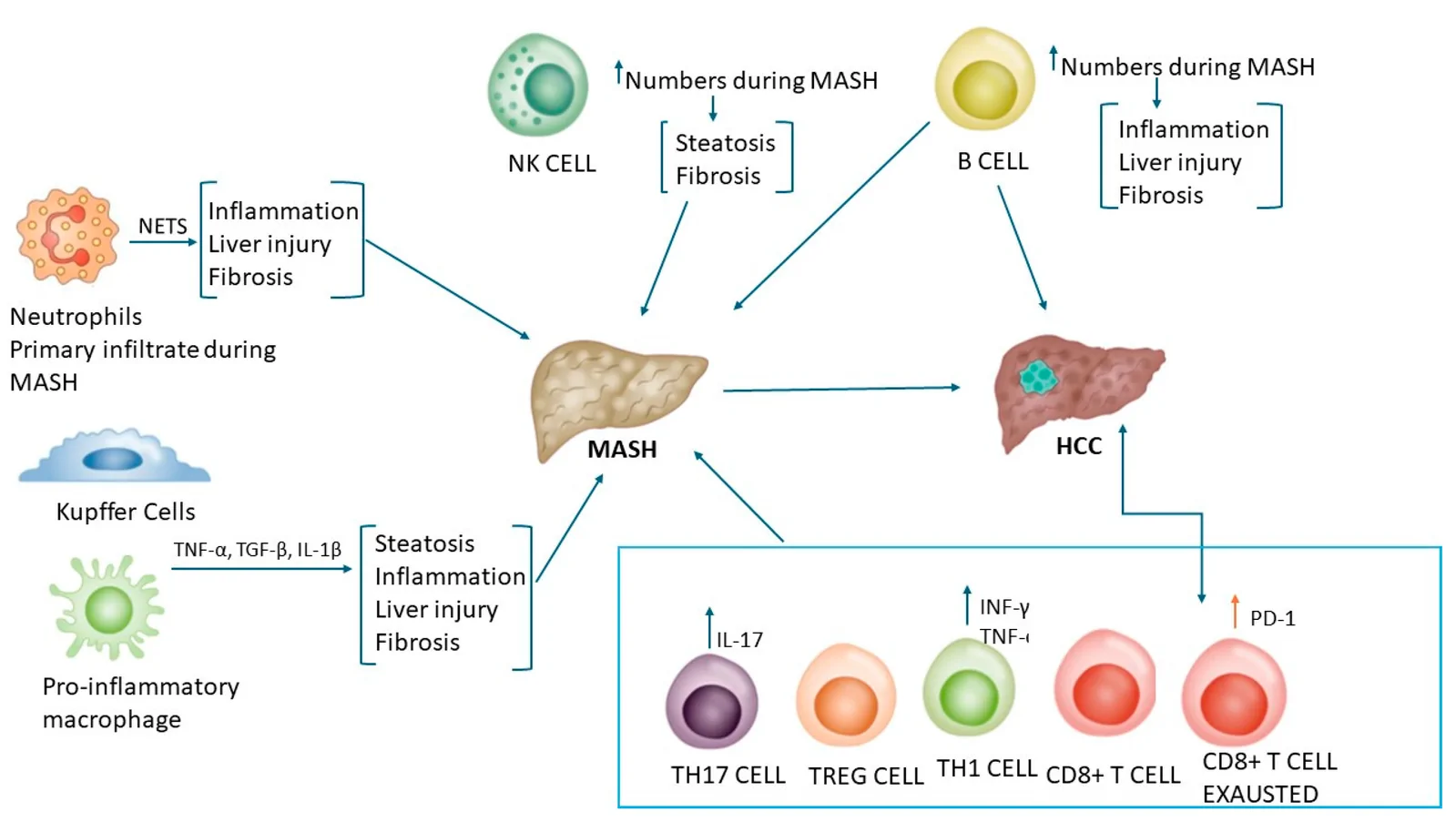
Schematic representation of immunity cells in MASLD and their role in promoting liver injury, fibrosis, and transition in HCC. A complex network and interactions between the liver’s resident cells, such as hepatocytes, Kupffer cells, and resident macrophages; immunity cells, such as neutrophils and B cells, involved in innate immune response; B cells; Th17 cells; Th1 cells; CD8^+^ cells; T Reg cells; and exhausted CD8^+^ T cells involved in adaptative immune response. Exhausted CD8^+^ cells are crucial players in the transition of MASH to HCC.

The T lymphocytes effectors of phagocytosis and of tumor killing [57] comprise three subgroups: CD4^+^ T cells (which include helper T (Th)1, Th2, Th17, follicular helper T (Tfh) cells, and T Reg cells), CD8^+^ T cells, and γδ-T cells (Figure 3) [58,59].

CD4^+^ T lymphocytes are preponderant effectors of adaptative response; they, in the presence of persistent pro-inflammatory stimuli, acquire a range of polarized phenotypes, which are mostly classified into Th1, Th2, Th17, and T Reg cells (Figure 3) [60,61,62].

Regarding the role of CD4^+^ T cells, it has been reported that Th1 and Th17 lymphocytes are prevalent in hepatic tissues and the peripherical blood of patients with MASH [63]. In MASH, Th1 cells are predominant and are induced by IFN-γ. Indeed, in IFN-γ- deficient mouse models of NASH, the inhibition of inflammatory response and the consequent protective effect against liver injury and hepatic fibrosis development have been reported [64].

T_H_17 cells have both homeostatic and pathogenic roles in producing cytokines such as IL-17F, IL-22, and IL-17A [65]. The latter plays an important role in MASLD progression; it has been reported that treatment with anti-IL-17 monoclonal antibody of high-fatty-diet (HFD) mice improved liver function with the attenuation of lipid accumulation. Moreover, the treatment was able to inhibit KC activation, inducing a decrement of pro-inflammatory cytokine levels [66].

An increase in the levels of Th17 has also been reported in MASH patients compared to those with MASLD [67]. Specifically, in MASH, the injured hepatocytes appear to be responsive to IL-17A signaling, upregulating the expression of its receptor IL-17RA, compared to healthy hepatocytes, where its expression is lower [68]. Several studies have reported that IL-17A can exacerbate steatosis and fibrosis by promoting hepatic DNA injury [68]. The prevalent role of Th7 signaling in MASLD progression has been confirmed in a model of HFD mice, where the administration of monoclonal antibody targeting IL-17 induced a marked decrease in hepatic lipid accumulation and attenuated liver fibrosis (Figure 2) [68].

CD8^+^ T cells mediate metabolic dysregulation and amplify the IR status [69]; their activation is supported by type I IFN [70].

In both humans and mouse models, the hepatic accumulation of CD8^+^ T lymphocytes during MASLD progression. In particular, it has been demonstrated that, in mice with MASH, CD8^+^ T cells induced liver fibrosis via the activation of HSCs and through the production of IL-10 and TNFα [71]. Furthermore, in response to metabolic stimuli, the CXCR6^+^ CD8^+^ T lymphocytes act as auto-aggressive cells in a MHC-class-I-independent manner, resulting in the upregulation of Fas ligand expression and subsequent hepatocyte apoptosis [10]. The pharmacologic or genetic ablation of CD8^+^ T cells ameliorates steatosis, IR, and inflammation (Figure 3) [72,73]. In mouse models, it has been shown that, in an inflammatory milieu, the losing of liver tolerance is mediated by KC activation and T Reg cell (T Reg cell) reduction (Figure 3) [25].

T Reg cells play an immunosuppressive role, contributing to liver tolerance in healthy liver [11]. In contrast to this, in MASLD it has been shown that T Reg cells are more susceptible to apoptosis in response to oxidative stress. Consequently, MASH patients showed a reduction in levels T Reg on the peripherical blood and in the liver, together with an imbalance between the intrahepatic levels of Th17 and T Reg cells. However, it is not clear if it is a consequence of local immune dysregulation or is mediated by extrahepatic factors (Figure 3) [67].

Immunological tolerance mechanisms include the expansion of CD4^+^ T Reg cells, as well as the apoptosis inhibition of autoreactive cells, a mechanism commonly described for CD8^+^ T cells.

The tolerance induction is related to the expression of the inhibitory molecule PD-L1, expressed on the surface of lymphocytes when the liver is healthy, whereas if it is injured, PD-L1 expression is suppressed, and the immunity system response acquires a phenotype influenced by Th1 cells. In this context, adaptive immunity mediates chronic and aggressive inflammation, inducing the increase inCD8^+^ T cytotoxic cell activation and the expression of the PD-1 receptor. Variations in the expression of inhibitory T cell receptors (PD-1 and 2B4) on CD8^+^ T lymphocytes have been linked to the immune tolerance necessary for the MASH progression [74].

## 3. Impact of PD-1/PD-L1 in Chronic Metabolic Diseases

### 3.1. The PD-1/PD-L1 Immune Pathway and the Role of CTLA-4/B7 and TIGIT/CD112 Co-Inhibitor Receptors

#### 3.1.1. PD-1/PD-L1 Immune Pathway

**Figure 4:**
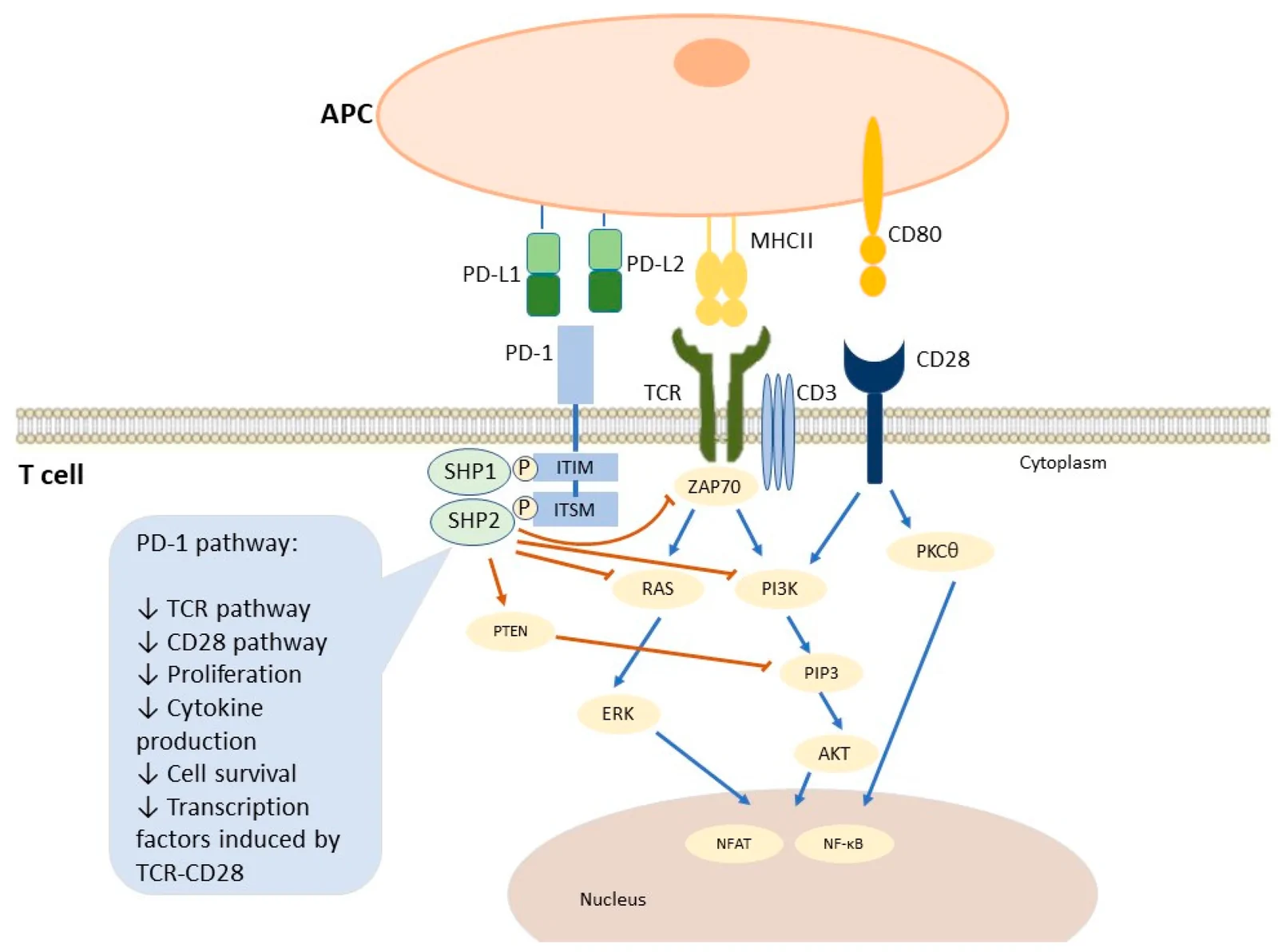
Schematic representation of PD-1 signaling. The interaction of PD-1 and PD-L1 or PD-L2 leads to tyrosine phosphorylation (P) of the immunoreceptor tyrosine-based inhibitory motif (ITIM) and immunoreceptor tyrosine-based switch Motif (ITSM). This phosphorylation recruits SHP-1 (Src homology phosphatase 1) and SHP-2 (Src homology phosphatase 2) that act on proximal signaling molecules and reinforce PTEN expression, thus effectively reducing the PI3K (phosphoinositide-3-kinase) and RAS (rat sarcoma) pathways. PD-1 signaling may result in decreased T-cell proliferation, survival, protein synthesis, and cytokine production. (Red arrows describe the inhibitory effect in PD-1-mediated signaling, whereas the blue arrow indicates the consequence of antagonist signaling CD80/CD28 of PD-1/PD-L1).

Programmed cell death protein 1 (PD-1), also called PDCD1 or CD279, is a member of the B7/CD28 receptor superfamily and is a type I transmembrane protein, with a complex organization into domains and into motifs, like ITIM and ITSM, required for its immunosuppressive activity [75,76] (Figure 4).

PD-1 is activated by two ligands, which are type I transmembrane proteins and belong to the B7/CD28 family: PD-L1, and PD-L2 [75,76,77]. They differ in their affinities for PD-1: PD-L2 has a higher affinity, though PD-L1 has a wider range of expression than PD-L2 [77]. PD-L1 and PD-L2 expressions are upregulated on T and B cells by the inflammatory cytokines type 1 and type 2 interferons, TNF-α, IL-2, IL-7, IL-21, and IL-15 [78,79]. In addition, cancer cells are expressed on both PD-L1 and PD-L2, but PD-L1 is the main player in tumor immune escape.

PD-1, a coinhibitory receptor, limits the over-activation of immune responses and promotes self-tolerance through the modulation of activated T cells, the induction of antigen-specific T cell apoptosis, and the inhibition of regulatory T cell apoptosis [78]. On the other hand, the PD-1 pathway limits the immunopathological responses; indeed, whether the response of CD8^+^ T cells is not adequately controlled, it can result in the overproduction of pro-inflammatory cytokines, driving aberrant cell killing, tissue damage, and the development of severe immunopathology.

In recent years, PD-1 has received much attention for its roles in inducing an exhausted T cell phenotype and tumor immunosuppression. Indeed, a correlation between PD-1 signaling and metabolic activity has been reported in T cells [80]. Specifically, during T cell activation, PD-1 signaling can modulate metabolic reprogramming, inducing the switch from oxidative phosphorylation to aerobic glycolysis, becoming effector cells [81]. In addition, in CD4^+^ T cells, the PD-1 pathway promotes lipolysis and fatty acid oxidation [82]. In the tumor microenvironment, this metabolic competition drives tumor progression by inducing the hyper-responsivity of T cells through glucose deprivation [83].

Several studies have reported PD-1 function in different cell types, but the knowledge of its signaling and functions on activated T cells is not completely understood. PD-1 mediates inhibitory signals when engaged with the T cell receptor (TCR) or the B cell receptor (BCR) (Figure 4) [84]. It is known that in T lymphocytes, PD-L1/PD-1 signaling antagonizes CD80-CD28 costimulation, inducing the inhibition of T-cell proliferation, cytokine production, and cytolytic function and impairing T-cell survival (Figure 4) [85].

PD-1/PD-L1 pathway may inhibit T-cell function and survival by directly blocking TCR signals or, alternately, can exert an indirect inhibitory effect on CD28-costimulation by regulating the expression of CK2 and cyclin-dependent kinases (CDKs) (Figure 4) [86].

#### 3.1.2. CTLA-4/B7: A Co-Inhibitor Receptor of Immune Response

The immune pathways are regulated not only by the PD-1/PD-L1 axis but by other ones, such as CTLA-4/B7 and TIGIT/CD112, which can contribute to the pathogenesis of MASLD and to the transitioning of HCC. CTLA-4/B7 (Cytotoxic T Lymphocyte Antigen 4) is an inhibitory immune checkpoint acting together with PD-1/PD-L1 in the regulation of physiological immune homeostasis. It downregulates inflammatory responses and can facilitate the immune evasion of cancer cells in the early stages of the disease [87]. CTLA-4 is expressed on CD4^+^, CD8^+^ T, and T Reg cells and negatively regulates their function, acting during their phases of activation. Indeed, differently from PD-1, which acts during the effector phase, impairing the T cells, proliferation and differentiation with the establishment of an exhausted phenotype, CTLA-4 is expressed on CD8^+^ and CD4^+^T cells after their stimulation, preventing the proliferation signal; conversely, it is expressed on T Reg cells in a constitutive manner [78,88,89]. CTLA-4 acts to recognizing ligands B7-1 and B7-2 (or CD80 and CD86), expressed principally on APC, and, respectively, in a manner constitutive of B7-1, whereas B7-2 acts later [90]. B7-1 and B7-2, expressed on APC, interact with the CD28 of T cells and induce the secretion of inflammatory cytokines, initiating inflammatory responses though the activation of CD8^+^, CD4^+^, and T Reg cells [91,92]. T Reg cells are considered canonical anti-inflammatory cells, and CD28 could, in this manner, indirectly contrast MASLD progression, activating hepatic T Reg accumulation.

On the other hand, when B7 interacts with CTLA-4, it induces the inhibition of naïve T lymphocytes and T Reg suppressive functions, resulting in the downregulation of the T cell response [93]. It has been reported that B7 costimulation (1 and 2) in mice fed HFD reduced the T Reg cell number, promoting MASLD progression [94].

In addition, it has been reported that, under conditions of low inflammation, such as in the early stages of tumorigenesis, APC cells express low levels of B7 molecules and CTLA-4 activity is lower, so it cannot inhibit the T cell response and allows them to participate in the antitumoral response [95]. In contrast, in conditions of chronic inflammation, during tumorigenesis progression, the activation of innate and adaptive immune cells can increase the expression of costimulatory molecules, such as B7, which, when interacting with CTLA-4, downregulates T cell activation [87]. In this manner, CTLA-4/B7 prevents the elimination of immune-mediated tumoral cells, favoring the development of immunological tolerance against them [96].

To date, immune checkpoint inhibitors (ICI) have been designed to be able to act against CTLA-4/B7 and PD-1/PD-L1pathways, and the efficacy of combined immunotherapy with MoAb in cancer treatment has recently been applied, showing higher clinical efficacy compared to either agent individually [97].

Since CTLA-4 plays a critical role in the downregulation of T cell responses at the beginning of the immune response, its inhibition, mediated by its blockade, could be a strategy for the enhancement of T cell responses in immunotherapy, especially in the early stage of tumorigenesis. Interest in these important immune checkpoint pathways arises from their role in modulating physiological immune homeostasis, downregulating the inflammatory response, and facilitating the immune evasion of tumor cells [98].

#### 3.1.3. TIGIT/CD112: A Co-Inhibitor Receptor of Immune Response

Another newly identified coinhibitory receptor is the T-cell Ig and ITIM domain (TIGIT) expressed by activated CD8^+^ T and CD4^+^ T cells, NK cells, T Regs cells, and the follicular T helper [99]. The ligands of TIGIT are CD112 and CD155, which are expressed by tumor cells and APC. CD155, also called Poliovirus Receptor (PVR), shows a high affinity for TIGIT, whereas CD112 is a low-affinity ligand for TIGIT biding. Interestingly, CD155 and CD112 are expressed by a variety of solid tumors, including liver cancer [100]. Recently, a novel coinhibitory receptor for T cells has been identified, named CD112R [101].

Similar to the CD28/CTLA4 pathway, TIGIT/CD112 constitutes an emerging signaling pathway relating to the regulation of T cell immunity; indeed, a TIGIT blockade increases CD8^+^ T cell expansion against tumor antigens [102]. In cancer, TIGIT is expressed, together with PD-1, on tumor antigen-specific CD8^+^ T cells and on exhausted CD8^+^ T cell subsets and is associated with constant inflammatory responses [103].

TIGIT acts to inhibit innate and adaptative immunity, indirectly blocking T cell function by binding to CD155 on DC and directly reducing T cell proliferation [104]. Indeed, TIGIT binds CD155 expressed on CD8^+^ T cells and NK cells, with higher affinity limiting their activation. In this manner, TIGIT impairs antitumoral immunity and sustains cancer development [105]. TIGIT blockade has been shown to increase antitumor NK cell activity and CD8^+^ T cell cytokine production/cytotoxicity; the antitumor T cell effect was NK-cell-dependent. In this context, the PD-1 and TIGIT blockades synergize to augment the proliferation and function of CD8^+^ T cells with antitumoral effects [106].

Considering the prevalent role of inhibitor receptors in regulating innate and adaptative immunity in chronic disease, such as MASLD, characterized by the hepatic progressive accumulation of exhausted CD8^+^ T cells, and in cancer, where the dysfunctional/exhausted T cells are prevalent, the upregulation of CTLA-4/b7 and TIGIT/CD112 checkpoint inhibitory receptors limits T cell survival and function. In this scenario, their interaction with the PD-1/PD-L1 pathway could amplify the mechanisms of immune escape, favoring MASLD progression or HCC development.

### 3.2. PD-1/PD-L1 Axis in Obesity

Obesity is a major risk factor for MASLD development and is responsible for low-grade inflammation in white adipose tissues (WATs) [107].

WAT plays an important role in energy storage and produces several biologically active molecules, such as adipokines, that act as regulators of systemic metabolism and immune modulators.

WAT is colonized by a wide range of leukocyte populations: CD4^+^, CD8^+^, Treg, B cells, iNKT, mast cells, eosinophils, innate lymphoid type 2 cells (ILC2s), macrophages, and DCs [107,108]. The resident adipose leukocyte disruption imbalances homeostasis, contributing to the development of obesity-associated inflammation and metabolic disorders.

In the initial stages of the inflammatory state, excessive nutrient intake causes the pathologic expansion of adipose tissue, which results in cell hypertrophy, the failure of lipid storage, hyperinsulinemia, dyslipidemia, and adipocyte hypoxia [109]. This persistent condition of oxidative stress and inflammatory stimuli leads to adipocyte apoptosis and the release of pro-inflammatory and chemotactic mediators and consequently leukocyte infiltration. Adipose inflammation leads to CD8^+^ T cell activation, which propagates inflammatory signals and shifts the M2 phenotype of macrophages into M1 [108].

The M1 cells form crown-like structures (CLSs) around the apoptotic adipocytes, resulting in WAT inflammation [110].

In inflamed adipose tissue, persistent T-cell antigen receptor stimulation induces CD8^+^ T cells to acquire an exhausted phenotype and consequently the loss of proliferative activity and cytotoxic functions. Eljaafari et al. have demonstrated that in mice and obese humans, the over-expression of PD-1 is responsible for exhausted T cells [111]. Shirakawa and colleagues demonstrated that in obese WATs, there is a unique subpopulation of CD153^+^, PD-1^+^, CD44hi, CD4^+^, and T lymphocytes that is linked with WAT inflammation and systemic insulin resistance [112]. In a murine model of obesity, PD-1 is an intrinsic negative regulator of ILC2 function in the presence of PD-L1^+^ macrophages. TNF seems to play a central role in the disruption of homeostasis by the PD-1/PD-L1 pathway, triggering IL-33, and M1 activation [113]. In a knockout mouse model, PD-L1 expression limits adipose tissue inflammation and obesity with the increase in Th1 cells and a reduction in ILC2 and Treg cells [114].

Obesity in NASH patients represent an important risk for HCC development. Recent studies have shown that cytokines produced by the inflammasome may contribute to the inflammatory microenvironment in obesity, which promotes NASH progression and HCC [115].

Data indicate that the NLRP3 inflammasome is stimulated by lipotoxicity and higher levels of IL-1β. In high-fat-fed (HFF) portal veins of mice, IL-1β is elevated [116].

### 3.3. PD-1/PD-L1 Axis in Type 2 Diabetes

Type 2 diabetes mellitus (T2D) is a complex chronic disorder that results from the dysregulation of lipids, protein, and carbohydrate metabolism, resulting in impaired insulin secretion and insulin resistance. A PD-1/PD-L1 signaling pathway is associated with T2D, which has proven to be a vital target for its therapy [117].

In patients with T2D, PD-1 is less expressed. It has been reported that the serum of these patients contains soluble costimulatory and cytokines, which influence PD-1 expression and function [118]. The induction of B-cell activation and cytokine production by follicular helper T cells (Tfh) is linked to the persistent low-grade inflammatory state that is characteristic of T2D. Indeed, in the evaluation of circulating CD4^+^CXCR5^+^ T cells (CTfh) in the peripheral blood of T2DM patients, a significant increase in the number of CTfh has been observed in the peripheral CD4^+^ T cells compared to controls. Additionally, an imbalance in the CTfh subtypes and an increase in the Th17 subtype have been reported in T2DM patients. Further analyses have revealed that CTfh levels were significantly high in patients with a body mass index (BMI) over 24.0. Interestingly, patients with abdominal obesity showed a further increase in CTfh levels compared to those without abdominal obesity. This finding suggests a potential association between CTfh and T2DM-related obesity. Another study has reported that PD-1 was more expressed in CD4^+^ T cells from healthy controls but not in T2D patients. A low expression level of PD-1 can result in abnormal T cells and the progression of T2D [119].

Nishimura et al. have demonstrated that CD8^+^ T cells are in charge of macrophage activation, and their polarization from M2 to M1 mediated by PD-1 triggers the progression of T2D into obese mice [108,120].

The number of NK cells has also been found to be higher in T2D patients [121]. T2D also impairs the function of NK, which is an important target for infection and tumor protection.

In tumor immunity, PD-1 upregulation can mediate the exhaustion of activated NK cells. PD-1 is expressed on NK cells in T2D patients and is less expressed in healthy donors. It has been reported that monocytes isolated from T2D patients have a low expression of PD-1, demonstrating that T2D patients display an altered insulin sensitivity [118].

### 3.4. The PD-1/PD-L1 Axis in MASLD

MASLD is a multifactorial disease, and its exact pathogenesis is not completely understood. Several interlinked processes contribute to inducing liver injury, steatosis, and inflammation, which together progress to fibrosis and, sometimes, to HCC. In recent years, PD-1 and PD-L1 checkpoint inhibitors have been used as therapy for HCC; indeed, immunotherapy is thought to activate T cells and reinvigorate immune surveillance against cancer. Recently, a relationship between the efficacy of immunotherapy and HCC etiologies has been underlined and consequently the importance of prognostic factors that allow an optimal response to therapy [122].

A meta-analysis study on the effect of inhibitors of PD-L1 or PD-1 in patients with HCC revealed that the immune therapy did not improve survival in patients with non-viral HCC. Moreover, patients with NASH-driven HCC who received anti-PD-1 or anti-PD-L1 treatment showed reduced survival compared to patients with other etiologies [122].

Recent data have reported that the hepatic progressive accumulation of unconventionally exhausted resident cytotoxic T cells (CD8^+^ PD1^+^ CXCR6^+^), which are characterized by a tissue residency (CXCR6), effector (granzyme), and exhausted (PD-1) phenotype [10].

**Figure 5:**
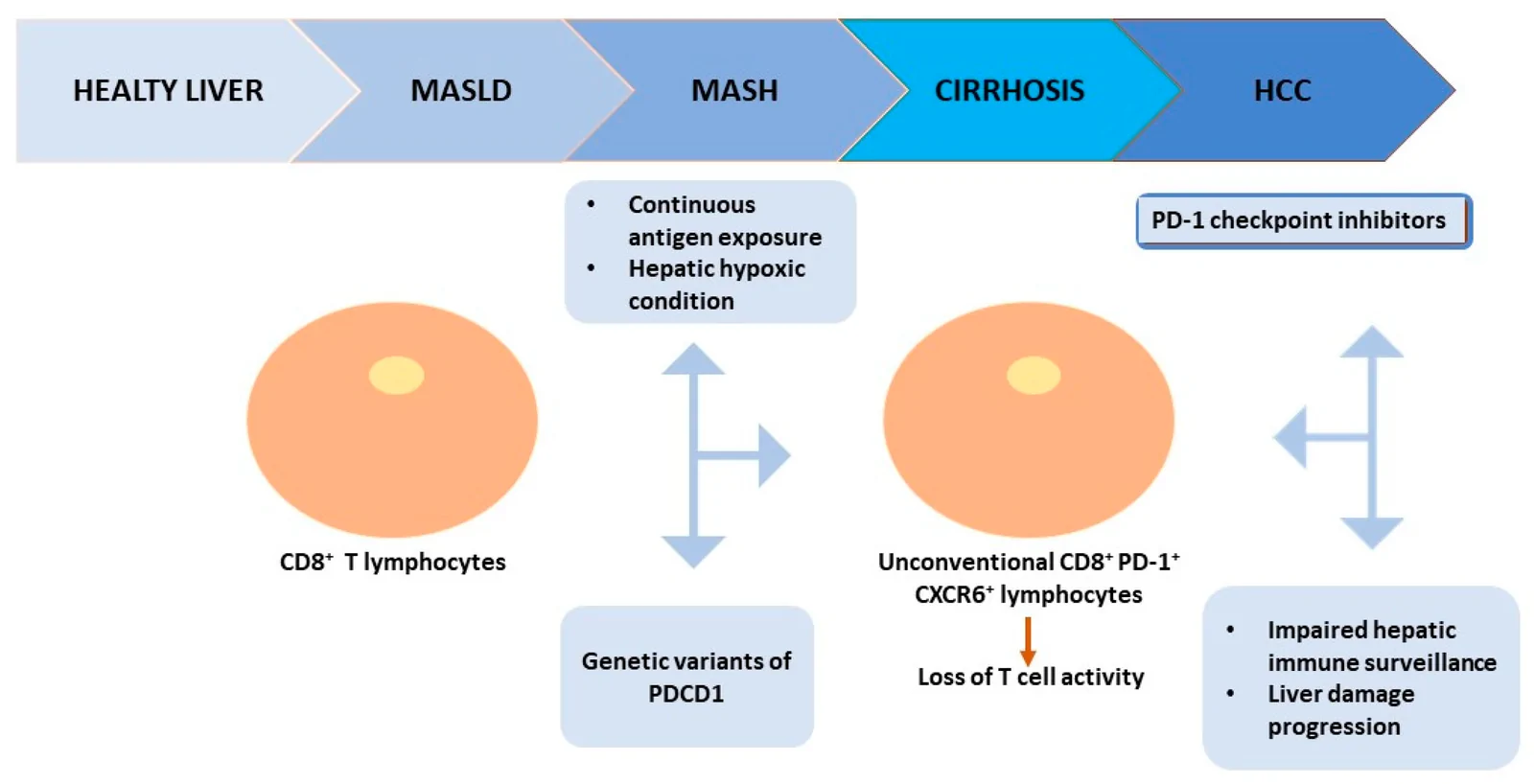
Schematic representation of MASLD pathogenesis and HCC transition. The accumulation of exhausted cytotoxic T cells is involved in the progression of liver disease. T lymphocyte activation and persistent expression of PD-1 are associated with the loss of T cell activity. CD8^+^ PD-1^+^CXCR6 lymphocytes impair immune surveillance, contributing to tissue damage and progression. Several single-nucleotide polymorphisms have been correlated with higher PD-1 expression in T lymphocytes.

The mechanism driving T cell exhaustion has been attributed to continuous antigen exposure, together with the hypoxic condition in the hepatic environment, leading to mitochondrial dysfunction [123]. Although the elimination of persistent stimulus can rescue terminally exhausted T lymphocytes, progenitor exhausted CD8^+^ T cells, which maintain their capacity to secrete inflammatory cytokines, maintain a molecular exhaustion sign (Figure 5).

Ongoing T lymphocyte activation and the concomitant persistent expression of PD-1, associated with exhausted phenotype, are related to the loss of T cell activity and tumor escape from immune responses.

Moreover, it has been observed in preclinical models and patients of MASLD or MASH treated with PD-1-direct antibodies that CD8^+^ PD1^+^ CXCR6^+^ lymphocytes showed impaired hepatic immune surveillance, and they also contribute to the progression of tissue damage [122] (Figure 3). Tissue damage was related to an increase in hepatic CD8^+^ PD1^+^ CXCR6^+^ T cells. Instead, the depletion or neutralization of CD8^+^ PD1^+^ CXCR6^+^ lymphocytes attenuates the progression of hepatic cancer.

Interestingly, in the setting of excessive fat accumulation and lipotoxicity, the PD1 checkpoint inhibitors treatment leads to an expansion of intra-tumoral T lymphocytes (TIL), exacerbating liver damage and promoting tumor progression. Fatty liver disease likely seems to inhibit immune responses against HCC.

In the setting of metabolic fatty liver disease, the crucial role of immune system dysregulation and, in particular, of cytotoxic lymphocytes has been highlighted; indeed, the hepatic accumulation of CD8 T cells with phenotypes that combined tissue residency (CXCR6) with effector (granzyme) and exhaustion (PD1) characteristics has been detected [10,122].

Several studies have reported the association of polymorphisms in PD-1 and PD-L1 genes with a higher risk of cancer development [124]. The correlation between the single-nucleotide polymorphism (SNP) rs7421861 of PDCD1 with higher PD-1 expression on monocytes and T lymphocytes has been demonstrated, demonstrating a potential role for PD1 in the susceptibility of MASLD to HCC [125,126].

#### Pro- and Anti-Inflammatory Effects of PD-1/PD-L1 Axis in MASLD Progression

The lipotoxicity-mediated damage to hepatocytes involves the activation of innate and adaptive immune responses. During the initial phase of inflammation, CD4^+^ effector T cells are activated first, and only later do NKT cells and CD8^+^ T cytotoxic lymphocytes contribute to tissue injury [34]. As a consequence of T cell activation, lymphocytes and liver cells overexpress PD-1 and PD-L1 as a self-defense mechanism to limit the activity of T cells [127].

The role of PD-1 in inflammation in MASLD and MASH is still not properly understood. However, even with the lack of published studies relating to its involvement, links from other inflammatory-related diseases and known inflammatory factors can be translated to MASLD and to MASH.

In the initial stage, chemokines and growth factors stimulate and activate T cells. However, chronic antigen exposure leads T cells to acquire an exhaustion phenotype with consequent PD-1 upregulation [85]. In this context, both CD4^+^ and CD8^+^ T cells can be differentiated in cellular subsets, contributing to the establishment of a pro- or anti-inflammatory status. Among these, Th1 and Th17 CD4^+^ T cells mediate a pro-inflammatory role, whereas Th22 and Treg CD4^+^ T cells play an anti-inflammatory role [22].

In the different phases of the inflammatory process, the upregulation of PD-1 in the Th1 or Th17 CD4^+^ T cells leads to the resolution of inflammation, whereas the overexpression of PD-1 in the Th22 or Treg CD4^+^ T cells amplifies the pro-inflammatory status, which, together with CD8^+^ T cells, is responsible for the progression from MASLD to NASH [63]. However, it has been reported that perforin-secreting CD8^+^ T cells protect against hepatic inflammation in mice with MASH.

On the other hand, the role of ligands of PD-1 and PD-L1 in MASLD and NASH is better understood. During the chronic lipotoxicity-mediated injury of hepatocytes, the activation of KCs is mediated by TLR receptors [128]. KCs represent the first line of defense and, when activated, they release pro-inflammatory factors, such as TNF-α, IL-1β, IL-6, IL-12, and IL-18 [21]. These cytokines and chemokines upregulate the expression of PD-L1 [97]. The overexpression of PD-L1 prolonged the inflammation within the liver, resulting in an upregulation of both PD-1 and PD-L1. The activation of PD-1/PD-L1 initially acts as a response to limit damage to the liver tissue caused by the immune system, but secondarily, their persistent activation contributes to the progression of MASLD.

## 4. Potential Role of PD-1/PD-L1 as Biomarkers in MASLD

No biomarkers related to the PD-1/PD-L1 axis are currently available for helping clinical practice, but the relevance of the PD-1/PD-L1 axis in the pathogenesis of MASLD suggests the intriguing possibility of using these molecules as biomarkers for the early identification of disease and for assessing the risk of progression, especially in the onset of HCC development in MASLD.

However, two major challenges should be addressed. First is the feasibility and reliability of measuring PD-1/PD-L1 expression in clinical settings. Second is how utilizing these biomarkers might impact early intervention strategies or patient management. A recent study [126] showed a lack of correlation between a PD1 expression and PD1 rs13023138 G variant. This may significantly complicate the search for reliable biomarkers. This may be particularly true when the involved variant is intronic, as not all genetic polymorphisms or intronic variants directly translate into relevant changes in protein expression or biological function. This scenario underscores the need for a more in-depth and detailed analysis of the different genetic variants involved in the PD-1/PD-L1 axis and their effect on protein expression and biological function to identify more reliable and clinically useful biomarkers for MASLD.

## 5. The Rationale of Immunotherapy in MASLD

The complex and central role of the immune system in the pathogenesis and progression of MASLD sheds light on the potential use of immunomodulation as a target therapy.

Although many drugs against MASLD are under investigation, no pharmacological therapy has been approved so far, and lifestyle changes play a dominant role in clinical practice [129].

The full spectrum of molecules acting as chemokine and nuclear receptors, tyrosine-kinases, and molecules expressed on the surface of immune-system cells are currently in use as a target therapy in MASLD. The chemokine (C-C motif) ligand (CCL) 2/C-C chemokine receptor 2 (CCR2) pathway recruits Ly-6Chi monocytes, and it encourages the phenotypic switch from Ly-6Chi macrophages to Ly-6C low macrophages that are the main source of MMPs promoting fibrosis resolution [130,131]. The chemokine (C-C motif) ligand (CCL) 2/C-C chemokine receptor 5 (CCR5) acts as a regulator of migration, activation, and proliferation of collagen-producing activated HSCs/myofibroblasts [132]. Cenicriviroc is an antagonist of CCR2/CCR5 chemokine receptors on pro-inflammatory monocytes. It showed an improvement in fibrosis in patients with histological MASLD versus a randomized vs. placebo, controlled, phase IIb clinical trial (CENTAUR trial). However, these results were not further confirmed in the randomized vs. placebo, controlled, phase III trial (AURORA trial), and the development of the drug was discontinued [133].

Peroxisome proliferator-activated receptors (PPARs), a subfamily of the NR1C nuclear receptors, are ligand-activated transcription factors. The three isotypes, PPARα (NR1C1), PPARδ (PPARβ or NR1C2), and PPARγ (NR1C3), have different tissue-distribution patterns and functions, playing a key role in the liver as regulators of glucose and lipid metabolisms, inflammation through macrophage regulation and fibrosis, keeping hepatic stellate cells quiescent [134]. Elafibranor (GFT505), a dual agonist of PPARα and PPARδ, was shown to be promising in the randomized versus placebo, controlled, phase IIb clinical trial (GOLDEN-505 trial). The post hoc analysis showed that the administration of 120 mg of Elafibranor resolved NASH without worsening fibrosis in the intention-to-treat analysis and in patients with moderate or severe MASH. However, the predefined endpoint was not met in the intention-to-treat population. However, Elafibranor failed to achieve the primary endpoint in the randomized versus placebo, controlled, phase III trial (RESOLVE-IT trial) [135]. Lanifibranor, a pan-PPAR agonist, resulted in a decrease of at least two points in the SAF-A score without a worsening of fibrosis compared to placebo in MASLD in a randomized, controlled, phase IIb clinical trial (NATIVE trial), supporting the further assessment of Lanifibranor in phase III trials [136].

Farnesoid X receptor (FXR) is a nuclear receptor expressed in the liver, with two known variants, FXRα (NR1H4) and FXRβ (NR1H5). Upon ligand activation, FXR binds to the transcriptional responsive elements as either monomers or heterodimers with retinoid X receptor (RXR), and it seems to exert an anti-inflammatory and anti-fibrotic effect and regulate glucose and lipid metabolism [129].

Obeticholic acid (OCA) is a 6α-ethyl derivative of chenodeoxycholic acid (CDCA) and represents the first-in-class selective steroid FXR agonist. It showed an improvement of fibrosis in histological MASLD in the randomized, controlled phase IIb clinical trial (FLINT trial) [137]. The randomized, controlled, phase III clinical trial (REGENERATE trial) revealed a potential effect of OCA on improving fibrosis compared to placebo but without any effect on MASH resolution, which did not encourage the approval of the drug by the FDA [138].

Tropifexor is an FXR agonist that was used alone and in combination with Cenicriviroc in histological MASH in a randomized versus placebo, controlled, phase IIb clinical trial (TANDEM trial). The safety profile of the combination was similar to respective monotherapies, and no substantial incremental efficacy was observed with the combination on ALT, body weight, or in histological end points compared with the monotherapy [139].

Monoclonal antibodies targeting T cell receptor-associated molecule CD3 represent a new horizon in MASLD treatment. However, the use of the murine monoclonal antibody (OKT3) is limited due to the toxicity shown in a phase IIa clinical trial, and a new fully human anti-CD3 antibody, Foralumab, already tested for Chron’s Disease and in renal allograft rejects, is going to be tested in a setting of MASLD and type 2 diabetes patients [140].

The spectrum of the potential treatment of MASLD through the immunomodulation is huge, but none of the treatments have been fully convincing so far. Further efforts are required to understand the pathogenesis and to control the progression of MASLD. Moreover, we cannot rule out the possibility of combination immunotherapy that might be the new frontier in MASLD treatment in the future.

## 6. Limitations

The clinical trials currently present in the literature demonstrate how the PD-1/PD-L1 axis could be used for the treatment of MASLD. However, the pathogenesis of MASLD is complex, involving multiple risk factors such as metabolic factors such as obesity and diabetes in a bidirectional manner, leading to lipotoxic damage, inflammatory response, and progressive deposition of fibrotic tissue. This could be a major limitation of immunotherapy, as it targets only a small fraction of the factors involved in the onset and progression of the disease, potentially accounting for the inadequate action of drugs tested thus far. Likely, a combination therapy might hold the key to optimizing the efficacy of drugs acting on the PD-1/PD-L1 axis. Although patients in such trials were correctly randomized, biases in patient’s enrollment, centralized biopsies reading, inadequate sample size, and short follow-up might influence the outcomes.

## 7. Conclusions

The dysregulation of the immune system plays a crucial role in the pathogenesis of MASLD and its progression to HCC.

In obesity and T2D, the PD-1/PD-L1 pathway mediates the dysfunction of hypertrophic adipocytes, M2-to-M1 polarization, and IL-33 production, maintaining inflammation of WAT, and inducing systemic insulin resistance. In MASLD progression, mitochondrial dysfunction and lipotoxicity upregulate PD-1, which results in the exhaustion of resident cytotoxic cells. In the tumoral context, PD-1-PD-L1 signaling mediates the exhaustion of activated NK cells and T lymphocytes. The continuous activation of T lymphocytes promotes tumor cells to escape from the immune response.

To date, it has been reported that immunotherapy improves survival only in patients with viral HCC compared to non-viral ones, whereas in MASLD-related HCC patients, the blockage of PD-1/PD-L1 signaling is associated with the decreased survival of patients.

PD-1/PD-L1 inhibitors play a critical role in cancer. Several monoclonal antibodies act as inhibitors to disrupt the interaction between PD-1 and PD-L1. In 2018, the FDA approved therapy, which only treats patients with metastasis or those who are not candidates for surgery or radiotherapy. Hence, studying the use of immunotherapy to block the PD-1/PD-L1 axis represents a complex goal for future study.

## 8. Future Directions

Although drugs targeting the PD-1/PD-L1 axis show significant promise in both MASLD and HCC, discussing future perspectives remains challenging. The pathogenesis of MASLD is complex and likely not entirely elucidated, and the treatment is equally complex and hardly standardizable. Large-scale population studies with follow-ups of at least three years are undeniably necessary to better understand the efficacy of these treatments.

As previously indicated, identifying optimal drug combinations targeting PD-1/PD-L1 and drugs with other mechanisms of action is crucial to develop therapies addressing multiple targets, potentially representing the future of MASLD.

## Data Availability

Not applicable.

## Abbreviations

APC	Antigen-presenting cell
CCR	Chemokine (C-C motif) ligand (CCL) 2/C-C chemokine receptor
CD	Cluster of differentiation
CTLA-4	Cytotoxic T Lymphocyte Antigen 4
DC	Dendritic cell
HCC	Hepatocellular carcinoma
HSC	Hepatic stellate cells
ICI	Immune Checkpoint Inhibitors
Ig	Immunoglobulin
ILC2	Innate lymphoid type 2 cell
IFN	Interferon
IL	Interleukin
IR	Insulin resistance
KC	Kupffer cell
LSEC	Liver sinusoidal endothelial cell
MoMf	Monocyte-derived macrophage
MASLD	Metabolic-associated fatty liver disease
MASH	Metabolic dysfunction-Associated Steatohepatitis
NASH	Nonalcoholic steatohepatitis
NETs	Neutrophil Extracellular Traps
NK	Natural killer
NKT	Natural killer T
OCA	Obeticholic acid
PPAR	Peroxisome proliferator-activated receptor
PD-1	Programmed cell death protein 1
PD-L1	Programmed cell death ligand 1
T2D	Type 2 diabetes mellitus
TGF	Transforming growth factor
TH	T helper
TIGIT	T-cell Ig and ITIM domain
TLR	Toll-like receptor
TNF	Tumor necrosis factor
T Reg	T regulatory
WAT	White adipose tissue
